# Body mass index contributes to sympathovagal imbalance in prehypertensives

**DOI:** 10.1186/1471-2261-12-54

**Published:** 2012-07-19

**Authors:** Gopal Krushna Pal, Adithan Chandrasekaran, Ananthanarayanan Palghat Hariharan, Tarun Kumar Dutta, Pravati Pal, Nivedita Nanda, Lalitha Venugopal

**Affiliations:** 1 Department of Physiology, Jawaharlal Institute of Post-graduate Medical Education and Research (JIPMER), Puducherry, 605 006, India; 2 Department of Pharmacology, JIPMER, Puducherry, 605 006, India; 3 Department of Biochemistry, JIPMER, Puducherry, 605 006, India; 4 Department of Medicine, JIPMER, Puducherry, 605 006, India; 5 Department of Biochemistry, Pondicherry Institute of Medical Sciences, Puducherry, 605 014, India; 6 Professor and Head, Department of Physiology, JIPMER, Puducherry, 605 006, India

**Keywords:** Prehypertension, Heart rate variability, Body mass index, Sympathovagal imbalance, LF-HF ratio

## Abstract

**Background:**

The present study was conducted to assess the nature of sympathovagal imbalance (SVI) in prehypertensives by short-term analysis of heart rate variability (HRV) to understand the alteration in autonomic modulation and the contribution of BMI to SVI in the genesis of prehypertension.

**Methods:**

Body mass index (BMI), basal heart rate (BHR), blood pressure (BP), rate pressure product (RPP) and HRV indices such as total power (TP), low-frequency power (LF), normalized LF (LFnu), high-frequency power (HF), normalized HF (HFnu), LF-HF ratio, mean heart rate (mean RR), square root of the mean squared differences of successive normal to normal intervals (RMSSD), standard deviation of normal to normal RR interval (SDNN), the number of interval differences of successive NN intervals greater than 50 ms (NN50) and the proportion derived by dividing NN50 by the total number of NN intervals (pNN50) were assessed in three groups of subjects: normotensives having normal BMI (Group 1), prehypertensives having normal BMI (Group 2) and prehypertensives having higher BMI (Group 3). SVI was assessed from LF-HF ratio and correlated with BMI, BHR, BP and RPP in all the groups by Pearson correlation. The contribution of BMI to SVI was assessed by multiple regression analysis.

**Results:**

LF and LFnu were significantly increased and HF and HFnu were significantly decreased in prehypertensive subjects in comparison to normotensive subjects and the magnitude of these changes was more prominent in subjects with higher BMI compared to that of normal BMI. LF-HF ratio, the sensitive indicator of sympathovagal balance had significant correlation with BMI (P = 0.000) and diastolic blood pressure (DBP) (P = 0.002) in prehypertensives. BMI was found to be an independent contributing factor to SVI (P = 0.001) in prehypertensives.

**Conclusions:**

It was concluded that autonomic imbalance in prehypertensives manifested in the form of increased sympathetic activity and vagal inhibition. In prehypertensives with higher BMI, vagal withdrawal was predominant than sympathetic overactivity. Magnitude of SVI (alteration in LF-HF ratio) was linked to changes in BMI and DBP. BMI had an independent influence on LF-HF ratio. It was advised that life-style modifications such as yoga and exercise would enable achieve the sympathovagal balance and blood pressure homeostasis in prehypertensives.

## Background

Hypertension is among the major risk factors for premature disability and death in both Western [1,2] and Asian [3] populations. Especially, Asian Indians have been reported to be at increased risk of diabetes, hypertension and heart diseases [4,5]. Though the increased risk of cardiovascular events in Indian population has been attributed to genetic predisposition [6], there is a strong correlation between changing lifestyle and increasing incidence of hypertension and heart diseases [7,8]. Studies have reported that sustained sympathetic overactivity, which increases vasoconstrictor tone of the systemic vasculature acts as a major mechanism for genesis of essential hypertension [9-11]. Though, several studies have revealed autonomic imbalance in hypertensive patients [12,13], there is paucity of data on the nature of autonomic imbalance that slowly leads to the progression from normotensive state into the state of prehypertension. Nevertheless, prehypertension has recently been reported to be associated with damage to the coronary vasculature and adverse cardiovascular events [14-16].

Recent reports from our laboratory based on the spectral analysis of heart rate variability (HRV) reveal that sympathovagal imbalance in the form of sympathetic overactivity and vagal withdrawal contributes to the development of prehypertension and hypertension in Indian population [17-19]. Our previous studies have revealed prehypertension is more prevalent among males and vagal withdrawal is more prominent compared to sympathetic overactivity in male prehypertensives [20,21]. We have also reported from the studies on young prehypertensives with parental history of hypertension that sympathovagal imbalance (SVI) is more intense in offspring of two parents hypertensive compared to the offspring of one parent hypertensive [22]. Yet, till date the exact mechanism of alteration in sympathovagal imbalance in prehypertensives has not been fully ascertained. Recently, contribution of obesity to the genesis of hypertension has been fully established [23]. However, no study has yet thoroughly evaluated the link of body mass index (BMI) with the causation of prehypertension. Spectral analysis of HRV has been used as a sensitive tool for assessment of autonomic dysfunctions in various clinical disorders [24]. Therefore, in the present study we have attempted to assess the nature and magnitude of autonomic imbalance and the role of BMI as an independent contributor to the genesis of SVI in prehypertensives.

## Methods

### Subjects

After obtaining the approval of Research Council and Institutional Ethics Committee, of Jawaharlal Institute of Post-graduate Medical Education and Research (JIPMER), Puducherry, India, 108 subjects were recruited from medicine out-patient department and staff of JIPMER. All the subjects underwent routine clinical examination to rule out the presence of any acute or chronic illness. It was ensured that all the subjects were healthy and physically fit to be included in the study. Eligible participants were interviewed and were explained about their participation and the nature of investigations to be conducted in the project. Informed written consent was obtained from all of them prior to the recordings.

The subjects between 25 to 45 years of age were included in the study. They were classified into following three groups based on their level of systolic and diastolic blood pressure as per JNC-7 classification [25] and the level of body mass index (BMI) as per the recommendation of world health organization (WHO) on BMI for Asian population [26].

1. Group 1 : Normotensive subjects with normal BMI (n = 45): Healthy subjects having systolic BP 100–119 mm Hg, diastolic BP 60–79 mmHg, and BMI 18.5-22.9.

2. Group 2 : Prehypertensive subjects with normal BMI (n = 27): Healthy subjects having systolic BP 120–139 mm Hg, diastolic BP 80–89 mmHg, and BMI 18.5-22.9.

3. Group 3 : Prehypertensive subjects with higher BMI (n = 36): Healthy subjects having systolic BP 120–139 mm Hg, diastolic BP 80–89 mmHg, and BMI 23 or above.

In the study population, there was no normotensive subject having higher BMI. Subjects were excluded from the study if they met any of the following criteria: (1) Subjects on antihypertensive therapy or receiving any medication, (2) subjects with history of smoking or alcoholism, (3) subjects with acute or chronic ailments, (4) subjects performing regular sports activities, and (5) known cases of diabetes mellitus, hypertension, myocardial infarction, heart failure, kidney disease or any endocrinal disorder. As the level of physical fitness is a major determinant of vagal tone [27,28], subjects performing regular athletic activities and body-building exercises were excluded from the study.

### Laboratory conditions and BP recording

Subjects were asked to report to autonomic function testing (AFT) laboratory of physiology department at about 9 AM following a light breakfast, without tea or coffee. The temperature of AFT laboratory was maintained at 25°C for all the recordings. After obtaining the informed consent, their age, height, body weight and body mass index were recorded. BP of all the subjects was recorded in AFT laboratory. Omron (SEM 1 Model), the automatic blood pressure monitor (Omron Healthcare Co. Ltd, Kyoto, Japan) was used for BP recording. The cuff size of Omron was 121 mm (width) × 446 mm (length), which was appropriate for all the subjects in the study. The length of the cuff tube was 600 mm. For BP recording, the subject was asked to sit upright with back straight on a wooden armed chair keeping one forearm on a wooden table kept in front and close to the subject. The height of the table was such that the middle of the arm placed on the table approximately coincided with the level of the heart. The subject was asked to keep the other forearm on the side hand rest of the chair. The BP cuff was tied just tight (neither too tight nor loose) on the arm approximately 2 cm above the cubital fossa. It was ensured that the BP cuff was at the level of the heart. After five minutes of rest in the same sitting posture, the ‘Start’ button of Omron was pressed that automatically inflated and deflated the cuff and SBP, DBP and basal heart rate (BHR) were noted from the display screen of the equipment. For each subject, SBP, DBP and BHR were recorded in each arm twice at an interval of five minutes between the recordings, and for each parameter the mean of the four recordings was considered. Rate pressure product (RPP) was calculated using the formula, RPP = systolic pressure × heart rate × 10^–2^[29].

### HRV recording

After 15 minutes of supine rest on a couch in AFT lab, ECG was recorded for 5 minutes for short-term HRV analysis following the standard procedures as practiced in the laboratory. For recording of HRV, recommendation of the Task Force on HRV was followed [30]. For the purpose, ECG electrodes were connected and Lead II ECG was acquired at a rate of 1000 samples/second during supine rest using BIOPAC MP 100 data acquisition system (BIOPAC Inc., USA). The data was transferred from BIOPAC to a windows-based PC with Acqknowledge software version 3.8.2. Ectopics and artefacts were removed from the recorded ECG. RR tachogram was extracted from the edited 256 sec ECG using the R wave detector in the Acqknowledge software and saved in ASC-II format which was later used offline for HRV analysis. HRV analysis was done using the HRV software version 1.1 (Bio-signal analysis group, Finland). Following frequency domain and time-domain indices were calculated from the HRV recordings.

A. Frequency domain indices (FDI)

1. Total power (TP)

2. Low frequency power (LF)

3. Normalized LF power (LFnu)

4. High frequency power (HF)

5. Normalized HF power (HFnu)

6. LF-HF ratio

B. Time-domain indices (TDI)

1. Mean heart rate (Mean RR)

2. Square root of the mean squared differences of successive normal to normal intervals (RMSSD) of HRV

3. Standard deviation of normal to normal interval (SDNN)

4. The number of interval differences of successive NN intervals greater than 50 ms (NN50)

5. The proportion derived by dividing NN50 by the total number of NN intervals (pNN50)

### Statistical analysis of data

SPSS version 13 (SPSS Software Inc., Chicago, IL, USA) and GraphPad InStat softwares (GraphPad Software Inc., San Diego, CA, USA) were used for statistical analysis. All the data were expressed as mean ± SD. Normality of data was tested by Kolmogorov Smironov test. For parametric data, the level of significance between the groups was tested by Student’s unpaired two-tailed ‘*t*’ test and for nonparametric data, the Welch’s corrected *t* test was used. Statistical analysis of data within the three groups was done by one-way ANOVA and post-hoc by Tukey-Krammer test. The association between LF-HF ratio and BMI with BHR, blood pressure and RPP was assessed by Pearson correlation analysis. The independent contribution of various factors such as age, BMI, BHR, SBP and DBP to sympathovagal imbalance (LF-HF ratio) was assessed by multiple regression analysis. The P values less than 0.05 was considered statistically significant.

## Results

There was no significant difference in age among all the three groups (Table 1). Though there was no significant difference in body weight and BMI between groups 1 and 2, body weight and BMI of group 3 were significantly increased compared to that of groups 1 and 2. Though the difference in BHR between groups 1 and 2 was not significant, BHR was significantly increased in group 3 compared to BHR of groups 1 and 2 (p < 0.001). SBP and DBP of groups 2 and 3 were significantly higher (p < 0.001) compared to that of group 1, though the difference was not significant between groups 2 and 3. RPP of groups 2 and 3 was significantly more compared to that of group 1 and RPP of group 3 was significantly more (p < 0.001) than that of group 2.

**Table 1:** Age, anthropometric and basal cardiovascular parameters in different groups

**Parameters**	**Group 1**	**Group 2**	**Group 3**	**p values**
	**(n = 45)**	**(n = 27)**	**(n = 36)**	
Age (Yrs)	35.70 ± 6.80	36.30 ± 7.25	37.12 ± 6.62	0.6521
Body Weight (Kg)	55.58 ± 4.56	56.04 ± 8.52	65.10 ± 6.20***,^##^	<0.000
BMI (Kg/m^2^)	21.16 ± 2.12	21.42 ± 1.78	27.80 ± 3.40***,^###^	<0.0001
BHR (per min)	69.80 ± 7.94	74.60 ± 7.30	85.10 ± 8.20***,^###^	<0.0001
SBP (mmHg)	108.65 ± 6.21	128.80 ± 7.20***	131.60 ± 8.25***	<0.0001
DBP (mmHg)	71.80 ± 5.45	84.56 ± 3.75***	85.76 ± 4.10***	<0.0001
RPP (mmHg/min)	73.70 ± 6.40	80.41 ± 7.32**	110.67 ± 7.92***,^###^	<0.0001

Data presented are mean ± SD; Group 1: Normotensive subjects with normal BMI; Group 2: Prehypertensive subjects with normal BMI; Group 3: Prehypertensive subjects with higher BMI; The * mark indicates comparison with normotensive subjects with normal BMI and the ^**#**^ mark indicates comparison with prehypertensive subjects with normal BMI. ** p <0.01; *** p <0.001; ^**##**^ p <0.01; ^**###**^ p <0.001.

**Figure 1:**
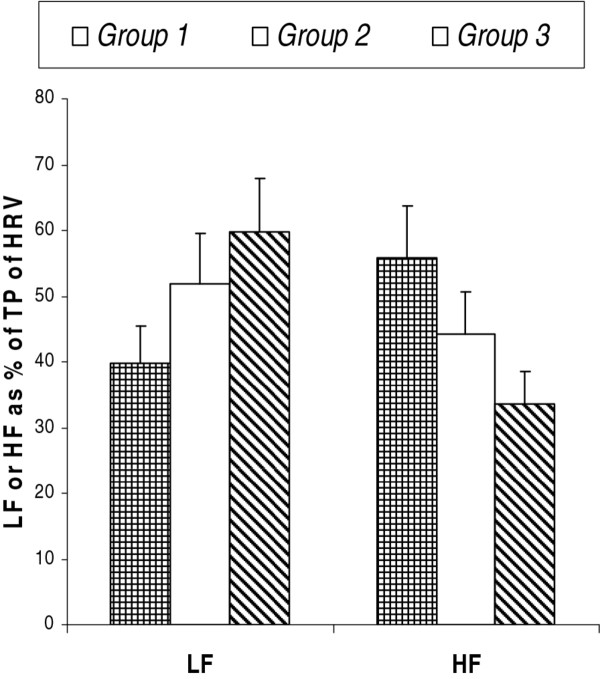
**Low frequency (LF) and high frequency (HF) powers as mean percentage of total power (TP) of heart rate variability (HRV) spectrum.** Group 1: Normotensives having normal BMI; Group 2: Prehypertensives having normal BMI; Group 3: Prehypertensives having higher BMI.

Total power (TP) of HRV spectrum of groups 2 and 3 was significantly decreased compared to that of group 1. Also, TP of group 3 was significantly less (p < 0.001) than that of group 2 (Table 2). Though there was no significant difference in LF power between groups 1 and 2, it was significantly less (p < 0.01) in group 3 compared to that of both groups 1 and 2. However, LF power expressed as % of TP was more in group 2 and 3 compared to that of group 1 (Figure 1). The HF power of groups 2 and 3 was significantly less (p < 0.001) than that of group 1, and HF power of group 3 was significantly less (p < 0.001) than that of group 2. Also, HF power expressed as % of TP was less in groups 2 and 3 compared to that of group 1 (Figure 1). Though LF_nu_ of group 2 (p < 0.05) and group 3 (p < 0.001) was significantly increased compared to that of group 1, change in LF_nu_ was not significant between groups 2 and 3. HF_nu_ of groups 2 and 3 was significantly low compared to that of group 1. Also, HF_nu_ was decreased significantly (p < 0.05) in group 3 compared to that of group 2. LH-HF ratio was increased significantly in groups 2 and 3 compared to that of group 1 and the LH-HF ratio of group 3 was significantly higher (p < 0.001) than the group 2 (Table 2).

**Table 2:** Frequency and time domain indices of HRV recorded in supine position of subjects in various groups

**Parameters**	**Group 1**	**Group 2**	**Group 3**	**p values**
	**(n = 45)**	**(n = 27)**	**(n = 36)**	
**FDI**				
TP (ms^2^)	980.80 ± 376.80	736.57 ± 245.60**	425.30 ± 186.40***,^###^	<0.0001
LF (ms^2^)	390.40 ± 186.70	382.64 ± 180.40	254.30 ± 130.50**,^##^	0.0006
HF (ms^2^)	548.30 ± 256.80	325.20 ± 136.10***	142.40 ± 78.90***,^###^	<0.0001
LF_nu_	40.32 ± 19.90	53.90 ± 20.30*	60.56 ± 24.70***	0.0004
HF_nu_	57.12 ± 21.80	46.05 ± 18.10*	35.28 ± 12.08***,^#^	<0.0001
LF:HF ratio	1.36 ± 0.65	2.34 ± 1.05**	3.76 ± 1.40***,^###^	<0.0001
**TDI**				
Mean RR (s)	0.859 ± 0.152	0.804 ± 0.140	0.700 ± 0.120 ***,^#^	<0.0001
RMSSD (ms)	34.10 ± 15.30	28.90 ± 11.30	19.25 ± 7.45***,^##^	<0.0001
SDNN (ms)	32.40 ± 17.20	28.50 ± 12.20	20.10 ± 9.20**,^#^	0.0005
NN50	26.40 ± 11.20	22.64 ± 10.70	17.05 ± 7.80***,^#^	0.0003
pNN50	8.90 ± 3.10	7.45 ± 2.50	5.10 ± 1.80***,^##^	<0.0001

Data presented are mean ± SD; Data of all parameters listed in this table are nonparametric except Mean RR. Group 1: Normotensive subjects with normal BMI; Group 2: Prehypertensive subjects with normal BMI; Group 3: Prehypertensive subjects with higher BMI; The * mark indicates comparison with normotensive subjects with normal BMI and the ^**#**^ mark indicates comparison with prehypertensive subjects with normal BMI. * p < 0.05; ** p <0.01; *** p <0.001; ^**#**^ p < 0.05; ^**##**^ p <0.01; ^**###**^ p <0.001.

Mean-RR, RMSSD, SDNN, NN50 and pNN50 were decreased significantly in group 3 compared to that of groups 1 and 2, though the difference in these parameters was not significant between groups 1 and 2 (Table 2).

Pearson correlation analysis was done to assess the strength of association of various factors with LF-HF ratio or BMI. In group 1, the correlation of LH-HF ratio was not significant with any of the parameters, and in groups 2 and 3, LF-HF ratio had significant correlation only with DBP (Table 3). As the association of BMI with LF-HF ratio was not found to be significant in groups 2 and 3, their correlation was assessed in the entire prehypertensive group. In all the prehypertensive subjects taken together, LF-HF ratio showed significant correlation with all the parameters except for age and SBP and correlation of BMI was significant with all the parameters except for age (Table 4).

**Table 3:** Pearson correlation of LF-HF ratio with age, BMI, basal heart rate, blood pressure and rate pressure product (RPP) of subjects of various groups

	**Group 1**		**Group 2**		**Group 3**	
	***r***	***p***	***r***	***p***	***r***	***p***
Age	0.072	0.683	0.171	0.393	0.050	0.774
BMI	0.121	0.490	0.156	0.438	0.230	0.295
BHR	0.087	0.620	0.202	0.311	0.234	0.170
SBP	0.131	0.452	0.113	0.575	0.017	0.921
DBP	0.117	0.505	0.542	0.003	0.456	0.009
RPP	0.085	0.629	0.186	0.354	0.207	0.225

The p values less than 0.05 was considered significant. Group 1: Normotensive subjects with normal BMI; Group 2: Prehypertensive subjects with normal BMI; Group 3: Prehypertensive subjects with higher BMI.

**Table 4:** Pearson correlation of LF-HF ratio and BMI with various parameters of all the prehypertensive subjects (n = 63)

	**LF-HF Ratio**		**BMI**	
	***r***	***p***	***r***	***p***
Age	0.034	0.789	0.160	0.211
BMI	0.543	0.000		
BHR	0.470	0.000	0.366	0.003
SBP	0.197	0.122	0.291	0.021
DBP	0.388	0.002	0.309	0.014
RPP	0.294	0.019	0.482	0.000
LF-HF ratio			0.543	0.000

The p values less than 0.05 was considered significant.

Multiple regression analysis was carried out to assess the independent contribution or link of various parameters to LF-HF ratio in prehypertensive population. BMI and BHR were found to have significant impact on LF-HF ratio (Table 5). BMI as an independent factor had maximum contribution (p = 0.001) to LF-HF ratio. Multiple regression analysis was not carried out for normotensive population, as the correlation of LF-HF ratio was not significant with any of the parameters in group 1 (Table 3).

**Table 5:** Multiple regression analysis of LF-HF ratio (as dependable variable) with various other associated factors (as independent variables) in the entire prehypertensive group (n = 63)

**Independent variables**	**Standardized regression coefficient B**	**95% C.I.**		**p values**
		**Lower bound**	**Upper bound**	
				
Age	0.018	−0.043	0.051	0.871
BMI	0.392	0.046	0.179	0.001
BHR	0.276	0.004	0.053	0.022
SBP	−0.036	−0.058	0.042	0.755
DBP	0.187	−0.015	0.158	0.101

p < 0.05 considered significant.

## Discussion

In the present study, significant decrease in total power (TP) of HRV spectrum in prehypertensive population (groups 2 and 3) compared to that of normotensive population (group 1) represents a substantial decrease in heart rate variability, which indicates decreased power of vagal drive in these subjects as TP in general reflects the vagal potency of cardiac modulation [24,30]. This was supported by a decrease in absolute HF power and HF_nu_ in prehypertensive subjects, as HF and HF_nu_ are the indices of parasympathetic drive to the heart [24,30]. Furthermore, TP, HF and HF_nu_ were significantly low in prehypertensive subjects with higher BMI (group 3) compared to the subjects of normal BMI (group 2) (Table 2) indicating the greater decrease in vagal drive in obese prehypertensive subjects.

A report by Wang et al has revealed increased sympathetic activity in prehypertensives [31], which was confirmed by our recent study [17]. In the present study we have observed a similar change in the form of increased LF_nu_ in prehypertensives compared to their normotensive counterparts (Table 2). Though there was an apparent decrease in absolute LF power in groups 2 and 3 (Table 2), which is due to the decrease in TP of HRV, LF when expressed as % of TP was much increased in these subjects (Figure 1). Thus, these findings confirm the increased state of sympathetic drive in prehypertensive subjects as LF represents sympathetic modulation [24,30]. LF-HF ratio is a sensitive measure of sympathovagal balance [24,30]. Increase in this ratio indicates increased sympathetic activity [24,30]. LF-HF ratio was significantly increased (p < 0.001) in prehypertensive subjects (groups 2 and 3) compared to the normotensive subjects (group 1), which indicates a heightened sympathetic discharge in prehypertensives. In the present study, in spite of the modest sample size, we found a big difference in LF-HF ratio between normotensive and prehypertensive groups, which was not observed in previous studies [32-34]. This difference in observation could be due to the racial difference in basal LF-HF ratio and the difference in altered cardiovascular autonomic tone in response to rise in blood pressure, supported by previous report on ethnic variation in HRV indices and the autonomic responses to stress [35]. However, till date, no report is available on the nature of alteration in sympathovagal balance that tilts towards augmented sympathetic activity in prehypertensives. Moreover, the contribution of altered vagal activity in the causation of prehypertension and hypertension has not yet been fully elucidated.

HRV has been used as a noninvasive tool to quantitatively estimate cardiac autonomic activity and it has proved to be of prognostic significance in hypertension [32-34]. HRV has been reported to be decreased in hypertension and the magnitude of decrease in HRV predicts the severity of hypertension [32]. HRV also has emerged as a cardiovascular risk marker [30]. It has been observed that among normotensive men, lower HRV has a greater risk for developing hypertension, and autonomic dysregulation has been documented in the early stage of hypertension [33]. Thus, decreased HRV in prehypertensives in the present study supports these earlier reports and could also be used as a predictive tool for the future development of hypertension in these subjects. Moreover, it was reported earlier that changes in cardiac autonomic function is associated with prevalent hypertension and reduced vagal activity along with the imbalance of sympathovagal function is associated with the risk of developing hypertension [34]. Therefore, the major findings of HRV analysis in the present study depicting decreased HRV, increased sympathetic and decreased parasympathetic drive indicate a similar pattern of alteration in sympathovagal balance in prehypertension as observed in hypertension earlier.

From the reports of the present study it appears that decreased vagal tone plays a critical role in the shift of this sympathovagal balance from the transition of normotensive state to prehypertensive state, as evident from decrease in TP and HF_nu_ in prehypertensives (Table 2). This is because the intensity of sympathovagal imbalance (degree of increase in LF-HF ratio) was more prominent in prehypertensive subjects with higher BMI compared to prehypertensive subjects with normal BMI. In prehypertensive subjects with higher BMI, though there was increased sympathetic activity (increased LF_nu_) and decreased parasympathetic activity (decreased HF_nu_), the magnitude of change in vagal drive was more than the change in sympathetic drive as there was significant difference in HF_nu_, but not LF_nu_ between the subjects of groups 2 and 3 (Table 2). Moreover, there was significant decrease in the all time domain indices (TDI) in group 3 subjects compared to that of both group 1 and 2 subjects, but not in group 2 subjects compared to the group 1 subjects. This indicates a profound decrease in vagal tone in prehypertensive subjects with higher BMI, as TDI in general reflect vagal modulation of cardiac activities [24,30]. Also, the basal heart rate was significantly more in prehypertensive subjects with higher BMI (group 3) compared to both the normal BMI subjects (groups 1 and 2), and there was no difference in BHR between normotensive subjects with normal BMI and prehypertensive subjects with normal BMI (Table 1). This indicates a significantly lower vagal tone in prehypertensive subjects with higher BMI as higher basal heart rate is an index of poor vagal tone [36]. Thus, these findings suggest that vagal withdrawal plays an important role in the alteration of sympathovagal balance with increase in BMI in prehypertensive subjects. Reports of our recent studies indicate significant contribution of vagal inhibition in the genesis of prehypertension in the young siblings of hypertensive parents [18,19]. Hence, we assume that vagal withdrawal could also be important in the causation of prehypertension in adults.

From this study the exact cause of sympathovagal imbalance in prehypertensive subjects can not be definitively ascertained. Nevertheless, BMI could be a potential factor for the causation of SVI as it was highly correlated with LF-HF ratio in prehypertensive subjects (Table 4). Moreover, BMI was strongly correlated with all the cardiovascular parameters including SBP and DBP in all prehypertensive subjects considered together (Table 4). In addition, BMI emerged as an important contributor to SVI in prehypertensives as it was found to have independent correlation with LF-HF ratio (Table 5) as determined by multiple regression analysis. Increased adiposity could be a key determinant for the development of prehypertension in susceptible individuals as obesity has been reported to be associated with increased sympathetic and decreased parasympathetic activity [37-40]. It was suggested that alteration in plasma levels of leptin, neuropeptide-Y and α-MSH (melanocyte-stimulating hormone) might be involved in activation of sympathetic activity that leads to hypertension in obese patients [41]. Further, there are report on cardiac dysfunctions associated with insulin resistance, oxidative stress and inflammation that are dependent on the quantity of fat mass in obese, but not in overweight children [42]. Therefore, we assume that SVI caused by increased adiposity is among the major predictors of increase in blood pressure in prehypertensives. This was further supported by the report of Schmid et al that increase in BMI is significantly associated with increase in sympathetic tone and increased blood pressure in young healthy overweight subjects [43]. Also, previous studies have demonstrated that sympathetic activity normalizes in overweight subjects following six-month calorie restriction combined with exercise [44].

Though the exact mechanism of increased blood pressure induced by sympathetic activation in obesity is not known, it has been suggested that retrograde inflammation could be the pathophysiologic link as increased sympathetic activity induces a proinflammatory state by IL-6 production, which in turn results in an acute phase response [45]. Also, increase in sympathetic activity has been reported to elevate blood pressure by promoting inflammation-mediated arterial rigidity in obesity [46]. As hyperinsulinemia is known to stimulate adrenergic activity and hyperinsulinemia is observed in obesity, it has been postulated that chronic hyperinsulinemia may lead to enhanced sympathetic tone and cardiovascular risk in obese patient [47]. Therefore, we propose to assess the link of BMI with inflammatory markers and hyperinsulinemia in the causation of SVI in prehypertensives in our future studies.

RPP is an indirect measure of myocardial load and oxygen consumption [29]. As degree of correlation of LF-HF ratio and BMI with RPP was highly significant in prehypertensives (Table 4), myocardial energy expenditure could be more in these subjects. Hence, the risk of cardiovascular dysfunctions associated with prehypertension [9-11] may possibly be linked to the level of BMI and RPP in these subjects.

Diastolic blood pressure (DBP) is the reflection of peripheral vascular tone and resistance [48]. In the present study, the degree of correlation of LF-HF ratio was maximum with DBP in groups 2 and 3 (Tables 3) and all the prehypertensive subjects taken together (Table 4). Hence, alteration in vascular tone could be directly linked to the degree and nature of SVI and it appears that BMI is an independent contributor to the genesis of SVI in these subjects. Inspite of the limitations that we have not performed direct assessment of sympathetic activity, not measured cardiac functions and there is less sample size of prehypertensive subjects having normal BMI, the present study emphasizes the necessity to improve vagal tone in individuals having blood pressure in prehypertensive range so that the sympathovagal balance is restored in these subjects and they do not progress into the stage of clinical hypertension. As practice of regular aerobic exercises such as morning walk, swimming, cycling etc. and practice of yoga such as asanas, meditation, pranayama etc. have been reported to decrease blood pressure, improve vagal tone and cardiac health, and reduce body weight gain [44,47,49-51], prehypertensive subjects should be encouraged to practice such program to prevent increase in their BMI and progression to hypertension.

## Conclusion

In the present study, sympathovagal imbalance (SVI) was observed in prehypertensive subjects, which was mild in subjects with normal BMI and severe in subjects with higher BMI. Vagal withdrawal was significantly associated with sympathetic overactivity in prehypertensive subjects. Magnitude of SVI (alteration in LF-HF ratio) was correlated with BMI and diastolic blood pressure. BMI had an independent contribution to change in LH-HF ratio. It was concluded that BMI could be a major predictor of SVI in prehypertensives. Hence, it was advised that life style modifications should be adopted by prehypertensives for achieving their effective autonomic homeostasis.

## Abbreviations

SVI, Sympathovagal imbalance; HRV, Heart rate variability; BMI, Body mass index; BHR, Basal heart rate; BP, Blood pressure; SBP, Systolic blood pressure; DBP, Diastolic blood pressure; JNC, Joint National Committee; TP, Total power of HRV; LF, Low frequency power; LFnu, Low frequency power normalized; HF, High frequency power; HFnu, High frequency power normalized; LF-HF ratio, Ratio of low frequency power to high frequency power; Mean RR, Mean RR interval depicting heart rate; RMSSD, Square root of the mean squared differences of successive normal to normal intervals; SDNN, Standard deviation of normal to normal interval; NN50, Number of interval differences of successive NN intervals greater than 50 ms pNN50 : Proportion derived by dividing NN50 by the total number of NN intervals; RPP, Rate pressure product.

## Competing interests

The authors declare that they have no competing interests.

## Authors’ contributions

AK: designed the study and drafted this manuscript, GKM: carried out the study and drafted the tables. RK: revising the manuscript procedures and drafting the manuscript, SS: helped in designing the study, MG: helped in drafting the manuscript and statistical analysis, AT: helped in designing and drafting the manuscript, FN: data analysis, NS: revising the manuscript. All authors read and approved the final manuscript.

## Pre-publication history

The pre-publication history for this paper can be accessed here:

http://www.biomedcentral.com/1471-2261/12/54/prepub
